# Social representations of Portuguese children about the coronavirus: a contribution to health education and developing life skills

**DOI:** 10.3389/fpubh.2026.1764010

**Published:** 2026-04-08

**Authors:** Carlos Alberto de Oliveira Magalhães Júnior, Leonardo Deosti, Fabiene Barbosa da Silva, Teresa Vilaça, Graça Simões Carvalho

**Affiliations:** 1 State University of Maringá, Maringá, PR, Brazil; 2 CIEC, University of Minho, Braga, Portugal

**Keywords:** central core, children’s drawings, health promotion, maximum similarity analyses, prototypical analysis

## Abstract

**Introduction:**

The COVID-19 pandemic has substantially affected daily life around the world. Social isolation, fear of infection, and discussion of protective measures were strongly conveyed in media and social discourse, including among children. Given this social dynamic, it is important to understand how the children’s Social Representations (SR) of coronavirus were formed so that health education strategies and life skills development can be devised.

**Objective:**

This study aimed to analyse the elements that potentially constitute the central core of Portuguese children’s SR about the coronavirus.

**Methods:**

Twenty-two children aged 9–12 years from a district in northern Portugal participated in the study. Data collection included administering a free word evocation test (FWET), which enabled interpretation through prototypical and maximum similarity analyses, and asking children to make drawings from the trigger term “coronavirus,” which were analysed using content analysis.

**Results:**

Based on the data, the elements that emerged as possible components of the central core of the children’s SR were “illness,” “death,” “masks” and “virus.”

**Conclusion:**

This study reinforces the importance of school health education combined with the media disseminating scientific and health information. It can also inspire and guide future work focused on children’s health education and life skills development.

## 1 Introduction

During the COVID-19 pandemic, digital media played a central role in shaping how the disease was understood, discussed, and responded to by different social groups. In this context, some people engaged in social movements that shared information leading to perceptions of COVID-19 vaccine threats and vaccine hesitancy (1). Indeed, although digital media served to disseminate measures to protect against the COVID-19 virus, it also provided a fertile ground for the spread of fake news about health and vaccination (2), as Gaspi and collaborators (3) point out: “the advent of social media has amplified movements that cast doubt on the efficacy of vaccination, promoting the spread of so-called ‘fake news’ through these platforms”.

It is important to highlight that the children were not merely passive recipients of information, but rather active social agents who constructed meanings about the coronavirus through their interactions with the media, family, and school. Therefore, there is a need for educational and communicative responses to the rapid spread of misinformation, through health education directed at young people (4). Schools are a key setting for developing children’s and adolescents’ life skills to enhance health education and improve school health promotion (5). Life skills development is recognised as a valuable tool in the service of education, health and social interaction (5). Thus, social interactions in the circulation of information about the COVID-19 pandemic can be shared, whether digitally or not, enabling the structuring of Social Representations (SR), as designated by Moscovici (6). These SR, in turn, permeate the subjects’ discourses, enable communication between individuals, and contribute to the construction of a shared reality (7).

The lockdown measures adopted in response to coronavirus pandemic altered social interactions and underscored the relevance of life skills in this context. In light of the foregoing, this study aims to analyse the elements that may constitute the central core of Portuguese children’s SR about the coronavirus, with the aim of informing school-based health education and life skills development. The decision to analyse children’s SR came from both the unique impact that the pandemic has had on this social group, due to social isolation and changes in their daily lives (8), and from the relevance of childhood as a period of intense meaning-making, in which SR can be formed and guide their social practices (9).

Several studies have examined children’s behaviour during the COVID-19 pandemic. For example, there is an analysis of the memories of children aged 8 to 16 years about their experiences over a year of the pandemic and how their psychological well-being changed during that period (10). In another study, Miao and collaborators (11) conducted a systematic review of longitudinal studies that addressed the mental health of children and adolescents during the COVID-19 pandemic, which allowed the identification of an increase in anxiety and depressive symptoms among young people. Despite these studies, there is still a gap in research on how children socially represent the coronavirus. In particular, no empirical studies were found that analyse the SR of COVID-19 in Portuguese children and their implications for health education and the development of life skills.

Despite this lack of research, the potential of such investigations can be observed in Iraklis (12) work, who conducted a study with Greek children aged 4 to 6 years using drawings accompanied by verbalisations. The study focused on understanding the representation of the COVID-19 virus and identified three main themes: the scientific knowledge children possessed about the virus; the perception of the virus as an enemy to be fought; and the understanding that confinement and isolation measures were necessary to prevent contamination. The findings of the cited work suggest that constructing this knowledge is necessary to propose post-pandemic health promotion strategies, build capacity to respond to future health and vaccination crises, and strengthen health literacy programs.

When research focuses on understanding the structure of SR, it is common to adopt a structuralist approach (09, 25), which has Jean-Claude Abric as its pathfinder. He developed the Central Core Theory (CCT), which describes that SR function as a system of interpretation of reality that governs people’s relationships with their physical and social environments, determining their behaviours and practices (13). According to the CCT, a SR consists of a central core (CC) and a peripheral system. The core is understood as the most stable and resistant part of the representation, whereas the peripheral system is flexible and variable (14). It adjusts itself and allows the contextualisation of a SR across different situations experienced by the social group. The peripheral system is composed of distinct zones that vary in their proximity to the central core and in their degree of stability (14).

Understanding the structure and functions of SR is essential for analysing how social groups construct shared meanings about phenomena, which in turn shape their practices and perspectives within a given social context. In this study, we adopt the structural approach to identify the central core and peripheral elements of Portuguese children’s SR about the coronavirus. Identifying the central core is particularly relevant for educational practice, as these elements organise meanings and orient behaviours, providing a theoretical basis for the design of school-based health education interventions aimed at promoting informed understanding and strengthening children’s life skills.

## 2 Methodology

This research is classified as mixed-methods, encompassing qualitative and quantitative analyses, offering both subjective and objective views of the data and thereby generating a broader understanding of the phenomenon under study (15). The structuralist approach to SR was adopted because it allows the central core and peripheral regions of the subjects’ SR to be identified, as proposed by the CCT (13).

The research subjects were 22 pupils from two fifth-year classes in two public primary schools located in different municipalities within the district of Braga, in northern Portugal. A convenience sampling strategy was adopted. The participants comprised 12 boys, 9 girls, and one child who chose not to identify their gender. In terms of age, they ranged from 9 to 12 years old (M = 10.33 years, SD = 0.73; one participant did not report age). The pupils from both classes were taught by a teacher who collaborated with the research and was responsible for data collection in her own classrooms. The schools and classes were selected based on accessibility and institutional collaboration, and all pupils in the selected classes were invited to participate. Participation was voluntary, and informed consent was obtained from the pupils’ legal guardians prior to data collection.

The choice of this age group was intentional, as children aged 9 to 12 years are in a developmental stage characterised by increasing cognitive, linguistic, and social abilities, which enable them to articulate meanings, engage in social dialogue, and construct shared understandings about socially relevant phenomena. In addition, children in this age range consciously experienced the COVID-19 pandemic and its impacts, such as school closures. Therefore, they represent an appropriate group for investigating SR about the coronavirus and its implications for health education and life skills development within the school context. It is also important to note that the data obtained in this study cannot be generalised, as they reflect the SR of a specific social group: children aged 9 to 12 years from public schools in northern Portugal. Even if data collection were conducted in another country with the same age group, the findings could be substantially different, given that the social contexts in which children are embedded—and, consequently, the media they are exposed to and the dialogues they engage in—may differ from those characterising the context of the present study.

The data were collected in 2024 through a questionnaire comprising three stages: (i) the first stage collected children’s personal information, such as gender, age, school, and city; (ii) in the second stage, the Free Word Evocation Technique (FWET) was used; and (iii) in the third and last stage the children expressed what they understood about the evocation term through drawings.

The FWET was adopted because it allows subjects to spontaneously express the terms that come to mind and are most accessible to their consciousness (16, 17) in response to an inducing term, in this research, the word “coronavirus.” Thus, children wrote down the first five words that came to their minds when they thought of the term coronavirus. Immediately afterwards, they were asked to rank these words by importance, with 1 being the most important and 5 the least. The process of ranking words is important because it allows subjects to rethink and reorganise the words evoked according to their relevance (18, 19). The term hierarchisation stage enables research participants to carry out a “[…] re-evaluation and subsequent cognitive reorganisation of the sequence in which the words were initially presented” (20). This procedure allows identification of which elements are most important to the participants and directly affects the allocation of evoked terms within the quadrants of the Vergès diagram, since the degree of importance attributed to the evoked terms is directly related to the calculation of the Mean Order of Evocation (MOE).

One of the procedures commonly included in the FWET is the request for justifications for each evoked word, which allows for a more in-depth semantic analysis and supports the categorisation of the terms. However, in this study, only six participants provided written justifications, which were extremely brief and insufficient for a consistent qualitative analysis. Therefore, to ensure equitable treatment of the data across participants, the justifications were excluded from the analytical procedures, and the categorisation of the evoked terms was based solely on lexical criteria.

In the third and final stage, the children expressed, through drawings, what they understood of the evocative term. Indeed, drawings are used in SR research because they allow subjects to express their ideas and feelings freely (21). Furthermore, it is a relevant technique for research involving children, as it can provide them with an opportunity to share their fears, feelings, and thoughts about sensitive issues (19).

Data collection was conducted in person on a single day, within the school setting, and on an individual basis, under the supervision of the previously mentioned collaborating teacher. The teacher received prior instructions regarding the data collection procedures and the administration of the activities. The entire data collection session lasted approximately two hours.

Before detailing the data analysis procedures, it is important to note that no pilot application of the instruments described was conducted. However, the instructions for the FWET and the drawing activity were previously standardised, following protocols well established in the literature (see, respectively, studies 09 and 03). Thus, these instruments are considered appropriate for the participant population of this study.

The data were analysed using a combination of analytical techniques: for data resulting from FWET, prototypical and similarity analyses were used; for the drawings, Bardin’s (22) Content Analysis (CA) was used, based on the work of Gaspi (19). Initially, the words evoked by the subjects were grouped according to lexical criteria, with words naming related elements placed in the same group (14). After this stage, the data analysis began.

Prototypical analysis is based on two criteria: the MOE of words and frequency (f) to construct the Vergès diagram (23), also known as the four-house diagram, which allows identification of the potentially central core and peripheral region of the SR. Frequency is calculated by summing the number of times the words in a group are repeated, while the MOE can be calculated using Equation 1.


$$\mathrm{MOE}=\frac{\mathrm{\Sigma G}}{f}\;\;\;$$ (1)

Where: ΣG is the sum of the degree of importance assigned to words and *f* is the frequency of evocation (14). After calculating the frequency and group MOE, the mean of these frequencies and MOE were calculated, where the Frequency mean (Fm) is given by the ratio between the sum of the frequencies and the number of groups, and the MOE mean (MOEm) by the ratio between the sum of the MOE and the number of groups (14). These mean values were adopted as cut-off points, in accordance with the structural approach of SR proposed by Vergès, allowing the distinction between high and low frequency and between early and late evocations.

Prototypical analysis uses quantitative indicators to organise the data. These indicators help researchers qualitatively interpret the SR structure. More specifically, “[…] the criteria of frequency and order of evocation complement each other and provide two collective indicators to characterise the salience of a word within a corpus generated by a group” (16). These metrics are used to allocate words to the quadrants of the Vergès diagram, thereby guiding the analysis. However, the definition of the structural role of the elements depends on a qualitative interpretation, grounded in the social context and in the theoretical framework adopted.

Based on these cut-off values, the Vergès diagram was constructed, following its predefined structure: groups with high frequency and low MOE were allocated to the first quadrant and represent the possible core of the SR; groups with high frequency and high MOE were part of the second quadrant; groups with low frequency and low MOE were in the third quadrant; and, finally, groups with low frequency and high MOE were in the fourth quadrant (14, 24). Based on the diagram, the elements potentially comprising the central core and the peripheral regions of the participants’ SR were identified, and reflections on them were outlined.

It is worth noting that, in this study, the analysis focused on elements potentially constituting the central core, as this component plays a key role in organising meaning within the SR, so that a change in the core automatically implies a change in the SR (24, 25).

The next step was a similarity analysis to complement the prototypical analysis and to strengthen the interpretation of elements potentially constituting the central core. To this end, the IRaMuTeQ (“Interface de R pour les Analyses Multidimensionnelles de Textes et de Questionnaire”) software was used to create the maximum similarity tree, where the circles represent the frequency of evocation of the groups, with larger circles indicating that the elements of this group were evoked more often (salience). The edges, or branches, connecting these circles represent the similarity index, i.e., how often two concepts were evoked simultaneously (24). In addition, the number of branches or connections a group has to other groups indicates its likelihood of belonging to the central core, as core elements tend to connect naturally with more elements (26). This analysis technique relates the number of lexical groups and the similarity index (14).

Similarity analysis also employs quantitative indicators to structure the data. In this case, indices of co-occurrence between evoked elements, the strength of the links, and the degree of connectivity among elements are considered. These parameters are calculated using IRaMuTeQ, are grounded in graph theory, and “[…] contribute to the identification of word co-occurrences which, in turn, reveal connections that facilitate the understanding of the structure of a text” (20). Although these indicators organise the data, the interpretation of the links between elements and their structural relevance depends on a qualitative reading guided by the theoretical framework of SR and by the conceptual contributions mobilised to understand the object under investigation.

**Figure 1:**
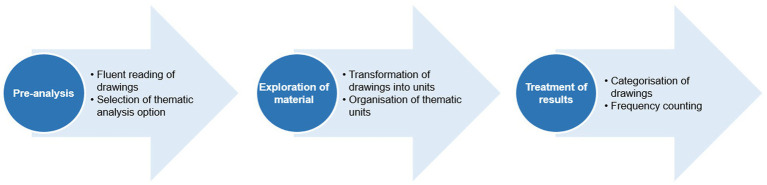
Process of analyzing drawings using CA. Source: Gaspi (21).

The final stage was the analysis of the drawings, in which a thematic categorical analysis of CA was performed, which consisted of discovering the ‘core of meaning’ (22) that make up communication and whose presence, or frequency of appearance, may mean something for the chosen analytical object. The stages defined by Bardin (22) were followed: (i) pre-analysis, (ii) exploration of material, and (iii) treatment of results, inference, and interpretation, taking into account the analysis of drawings carried out by Gaspi (19) as summarised in Figure 1.

In the first stage, a more general analysis of the drawings was carried out, separating them by theme and organising them into recording units. In the second stage, categories and subcategories for analysis were created based on the recording units. Finally, the categories were counted and, based on this, inferences and interpretations of the results were made (19, 22, 27). We underline that the drawings were analysed jointly by two of the authors. An inductive coding approach was adopted, involving the identification, analysis, and discussion of the elements present in the children’s drawings. This collaborative process aimed to achieve the consensual construction of categories. As the analysis was conducted through a consensus-based approach rather than independent coding, interrater agreement indices, such as Cohen’s Kappa, were not applicable.

It is worth noting that, according to the CCT, the identification of central elements through structural analyses should be understood as hypothetical and requires complementary methods for confirmation. Accordingly, the present study focuses on identifying elements that may constitute the central core rather than asserting its definitive composition.

Finally, regarding ethical and legal aspects, it should be noted that this study is part of a research project developed within a Brazilian higher education institution. The project was submitted to and approved by the Permanent Committee for Ethics in Research with Human Beings at the State University of Maringá, under Opinion No. 6,946,568 and Certificate of Ethical Appraisal No. 79619724. 3.0000.0104. As previously mentioned, data collection was authorised by the participating institutions where the collaborating teacher works, and informed consent was obtained from the children’s parents or legal guardians prior to data collection. As researchers responsible for the study, we ensured the anonymity of the participants and the confidentiality of the data. We also ensured that the data collected is used exclusively for research purposes.

## 3 Results and discussion

With the collected data, the first step was to group the 104 words evoked by the children into lexical groups. Although we asked each participant to evoke five words related to the trigger term “coronavirus,” two evocations were discarded because they did not fit into any of the 15 groups formed; two children evoked 4 words, and another evoked only 3.

The calculations described in the Methodology were also performed to obtain the f and MOE values for each lexical group and their averages. Thus, 5.93 was obtained for Fm and 3.01 for MOEm. The Vergès Diagram was then created, as shown in Table 1.

**Table 1:** The Vergès diagram with the distribution of lexical groups based on the children’s evocations about “coronavirus”.

Word	f	OME
Central elements - 1st quadrant		
High f and low mean order of evocations *f* ≥ 5.93 e MOE < 3.01		
Illness	20	2.35
Death	9	2.66
Virus	7	1.00
Intermediate elements - 2nd quadrant		
High f and high mean order of evocations *f* ≥ 5.93 e OME ≥ 3.01		
Masks	11	3.64
Pandemic	9	3.44
Hospital	8	3.25
Intermediate elements - 3rd quadrant		
Low f and low mean order of evocations *f* < 5.93 e OME < 3.01		
Cough	4	2.75
Lockdown	3	2.66
Hand sanitiser	2	1.50
Sadness	2	2.00
Symptoms	2	3.00
Peripheral elements - 4th quadrant		
Low f and high mean order of evocations *f* < 5.93 e OME ≥ 3.01		
Danger	4	3.25
Depression	3	4.33
Flu	3	4.33
Vaccines	2	5.00

Regarding the prototypical analysis, the results indicate that the elements that may comprise the core of children’s rational responses to coronavirus are “disease,” “death,” and “virus.” Commonly, in the application of the TALP (Test of Learning to Read), research participants are asked to justify their choice of words through short texts. However, in this research, only 6 of the 22 students completed this step. Therefore, to standardise data treatment, these justifications were disregarded.

Given the evocations, it is possible to infer that the students understand that coronavirus infection can progress to illness and that this can lead to death, demonstrating an understanding of how the infection occurs and the dangers it poses. This observation corroborates the findings of Folino and collaborators (28), who reported that children in Rio de Janeiro were aware of the risks of coronavirus contamination, the importance of preventive care, and, in some cases, the association between infection and death.

It is also possible to identify similarities with the work of Figueiredo and collaborators (29), who observed a high level of health literacy among students regarding COVID-19. In that study, the research participants demonstrated knowledge of the virus’s effects and recognition of the importance of adopting measures to prevent infection, reflecting knowledge and awareness of the importance of safe behaviours, mainly arising from media coverage of the pandemic and perceptions of its potential severity (29).

Three other groups that stand out are those in the second quadrant (“masks,” “pandemic,” and “hospital”), which have a high frequency but a lower OME than OMEm. This configuration indicates that these elements may form the central core of some children’s SR, but they are not sufficiently representative to constitute the central core of the whole children’s social group.

A final highlight is made for the elements’ vaccines’ and ‘flu,’ which make up the last quadrant. Although the data were collected after vaccination, and some of these students or their family members likely contracted the virus at some point, these terms are not structured as nuclear elements of their SR; however, they are present on the periphery, indicating greater fragility and a tendency to change over time.

**Figure 2:**
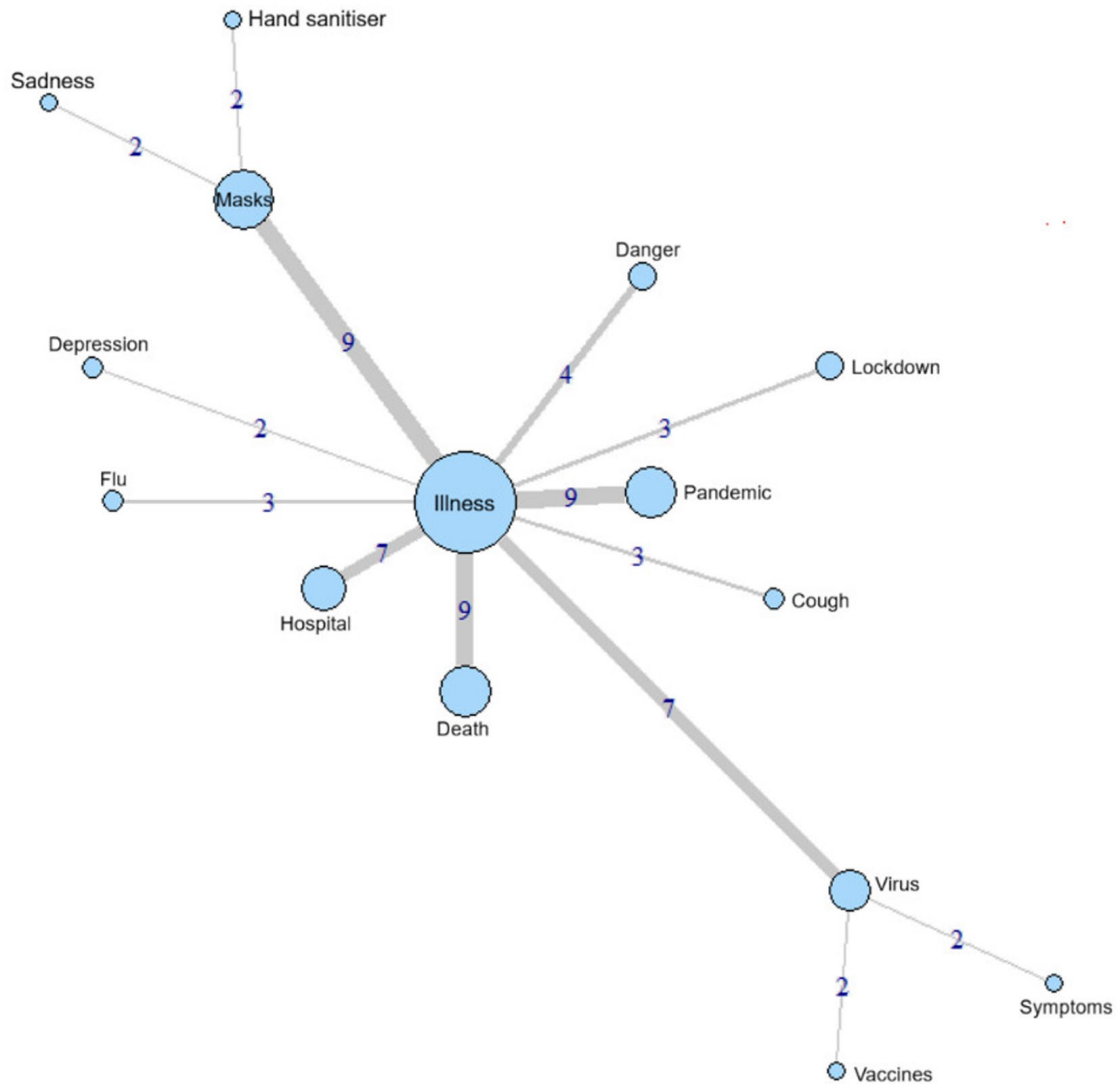
Maximum similarity tree of children’s evocations about “coronavirus”.

Although the prototypical analysis has provided clues about the possible elements that comprise the children’s RS central core, it is common to use similarity analysis to confirm these elements (14, 19, 30). Thus, using the IRaMuTeQ software, the maximum similarity tree was constructed and is shown in Figure 2.

The similarity analysis reveals that the elements “illness,” ‘masks,” and “virus” stand out as centralising groups, with a greater number of other branches originating from them. Ten branches stem from the “Illness” group, five of which have a high similarity index, while three branches stem from the “masks” and “virus” groups, one of which has a high index. Thus, it is possible to state that these elements are possibly part of the central core of the children’s SR, since the greater the number of branches emanating from an element (connectivity), the greater the probability that it belongs to the core, as core elements connect with a greater number of other elements in a natural way, i.e., they have a strong associative power (31).

The maximum similarity tree (Figure 2) corroborates the Vergès Diagram (Table 1) and confirms the possible children’s RS central core. In the prototypical analysis, the elements “illness” and “virus” are in the first quadrant of the diagram, whereas in the similarity analysis, they appear as centralisers.

Although there is a divergence regarding the element “death”: in the Vergès Diagram, it is in the central core (Table 1), but in the maximum similarity tree (Figure 2), it cannot be characterised as a component of the central core because it is not linked to several other elements. Furthermore, the term “masks” was placed in the second quadrant in the prototypical analysis (Table 1), whereas it took a central role in the maximum similarity tree (Figure 2). The divergence in interpretation of these terms allows for a critical discussion of the media’s role in the pandemic context. At that time, the constant dissemination of updated infection and death numbers across various media outlets was common. The intense circulation of the term “death” within this communicational context contributed to its early evocation by children, configuring it as a highly salient element in their SR.

Nevertheless, the term “death” does not perform the same organising function for practices and lived experiences as the term “masks,” since the latter is directly related to children’s everyday experiences during the pandemic and carries a practical, concrete, and experiential meaning. Wearing masks became an action incorporated into children’s school, family, and social routines, which favoured its articulation with a greater number of elements within the representational field. Thus, “masks” assumes the role of a practical organiser of the meanings attributed to the pandemic, whereas “death” constitutes a socially strong and widely disseminated symbol, but one with a lower relational capacity within the structure of children’s SR.

As shown in the methodological section, data triangulation was performed using prototypical, similarity, and CA. After interpreting the findings from the first two analyses, the description of what was observed in the CA begins. Two main themes were extracted from the children’s drawings (“actions aimed at protection” and “lack of care”) following the CA interpretation process described by Gaspi (19). The first theme consisted of categories that demonstrate individual, collective, or emotional elements shown in the children’s drawings. The second theme was formulated from a single category, focusing on the process of viral contamination and its consequences. Table 2 shows how the subcategories and categories were distributed within the aforementioned themes.

**Table 2:** Thematic categorical analysis of children’s drawings.

Theme	Category	Subcategory	Frequency
Actions aimed at protection	Devices for individual care	Masks	5
		Hand sanitiser	1
		Gloves	1
	Collective prevention measures	Blockage	2
		Lockdown	1
	Emotions	Apathy	1
		Sadness	1
		Aggressiveness	7
		Happiness	2
Lack of care	Contamination	Death	2
		Hospital	2
		Virus	19
		Droplets	2
		Illness	2

**Figure 3:**
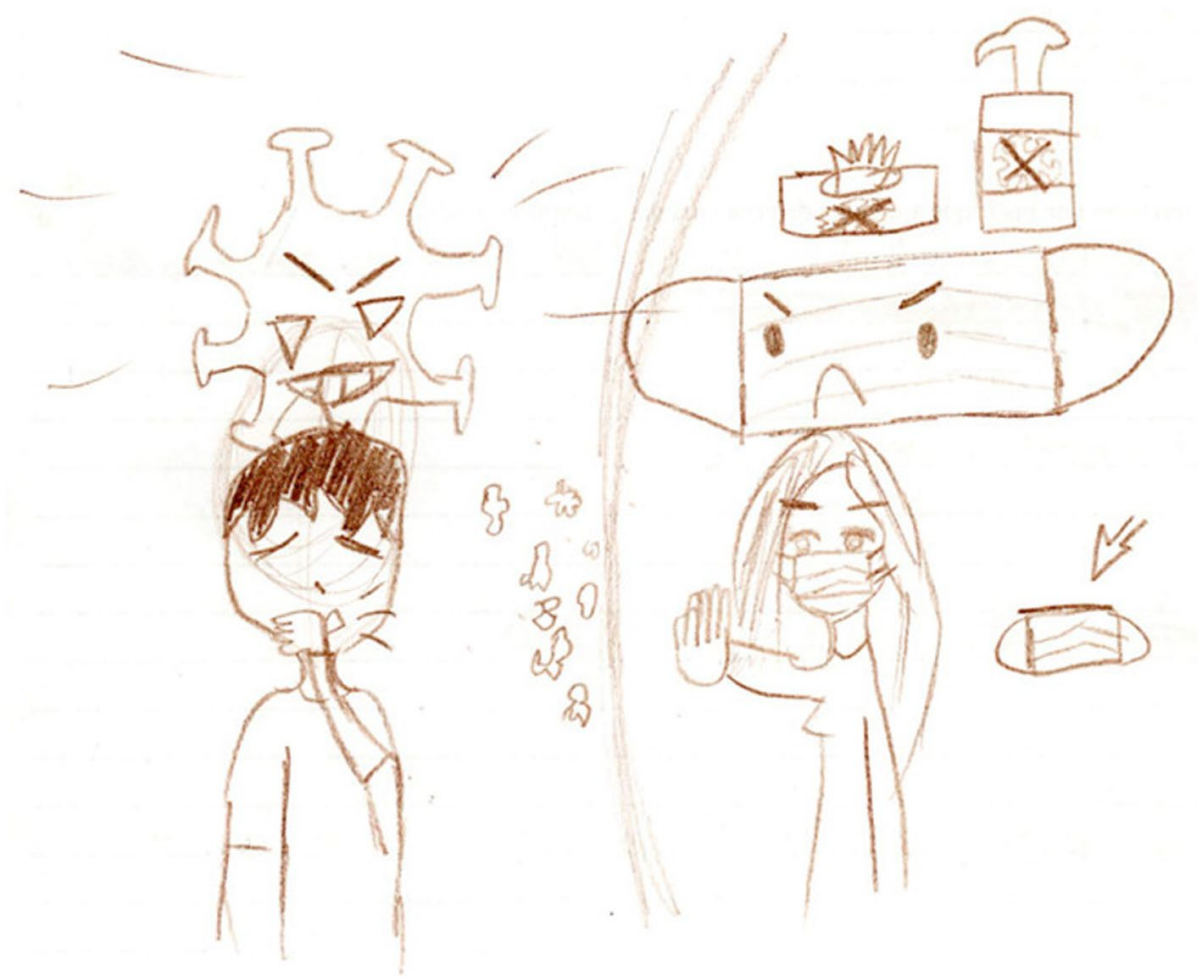
A child’s drawing made in response to the trigger term “coronavirus,” included in the subcategory “devices for individual care”.

The first category, “devices for individual care”, of the theme “actions aimed at protection” comprises three subcategories: “masks”, “hand sanitiser”, and “gloves” (Table 2). The presence of these elements in the children’s drawings shows that they understand and can illustrate the importance of these mechanisms as a strategy for preventing viral contamination. Figure 3 shows an example of a children’s drawing that includes these three elements.

Figure 3 shows several mechanisms of viral protection. The child was able to elucidate that the mask acts as a shield, preventing saliva droplets from being expelled into the air and carrying the virus. In addition, the child was also able to indicate the use of hand sanitiser and gloves to strengthen protection. This visual representation corroborates previous results by Gaspi (19), who shows children’s awareness of preventive measures. She also points out that the development of this awareness may be related to the dissemination of widely publicised information during the pandemic. This dissemination of information is based on the role of the mass media, which, according to Anwar and collaborators (32), is essential for promoting health education. These authors also argue the need for the media to disseminate public knowledge about maintaining a distance of 2 m, the proper use of face masks, and appropriate guidelines for recognising, diagnosing, and treating the disease (33).

**Figure 4:**
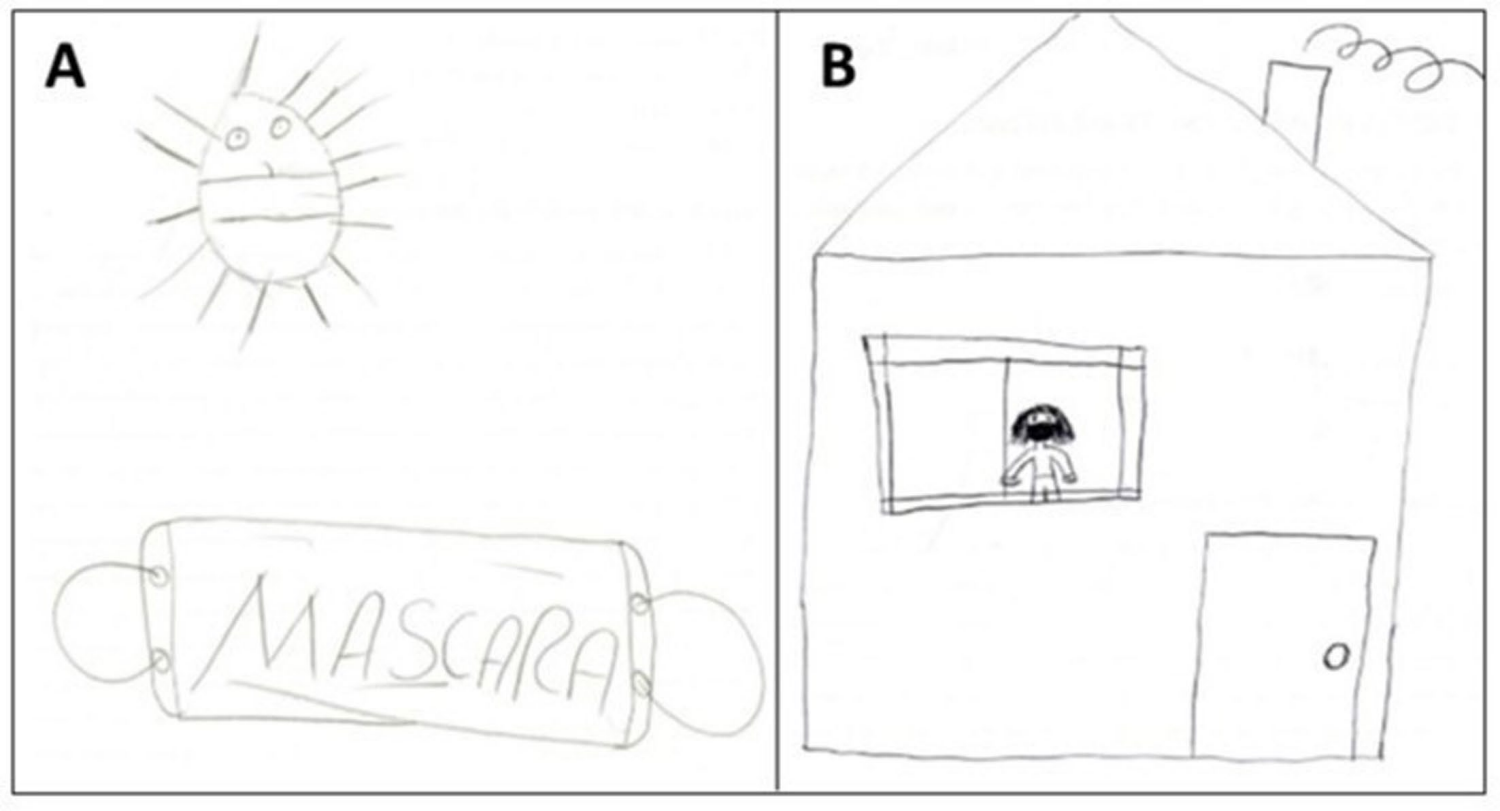
Two children’s drawings made in response to the trigger term “coronavirus,” included in the subcategory “collective prevention measures”: “blockage” **(A)** and “lockdown” **(B)**.

The “Collective prevention measures” category under the same theme (“actions aimed at protection”) comprises two subcategories, “blockage” and “lockdown” (Table 2 and Figure 4).

Two children’s drawings in Figures 4A,B show that both associated the use of a mask with a barrier. Therefore, the mask is shown to stop the virus from “biting” a person and thereby prevent human infection. The second drawing (Figure 4B) shows a person in lockdown, at home. The lockdown was a hard period, particularly for children, who, although understanding the importance of quarantine/isolation to stop the epidemic, most of these Portuguese age children (57%) were shown to be in favour of returning to face-to-face classes because it would enable better academic performance than at home (29).

**Figure 5:**
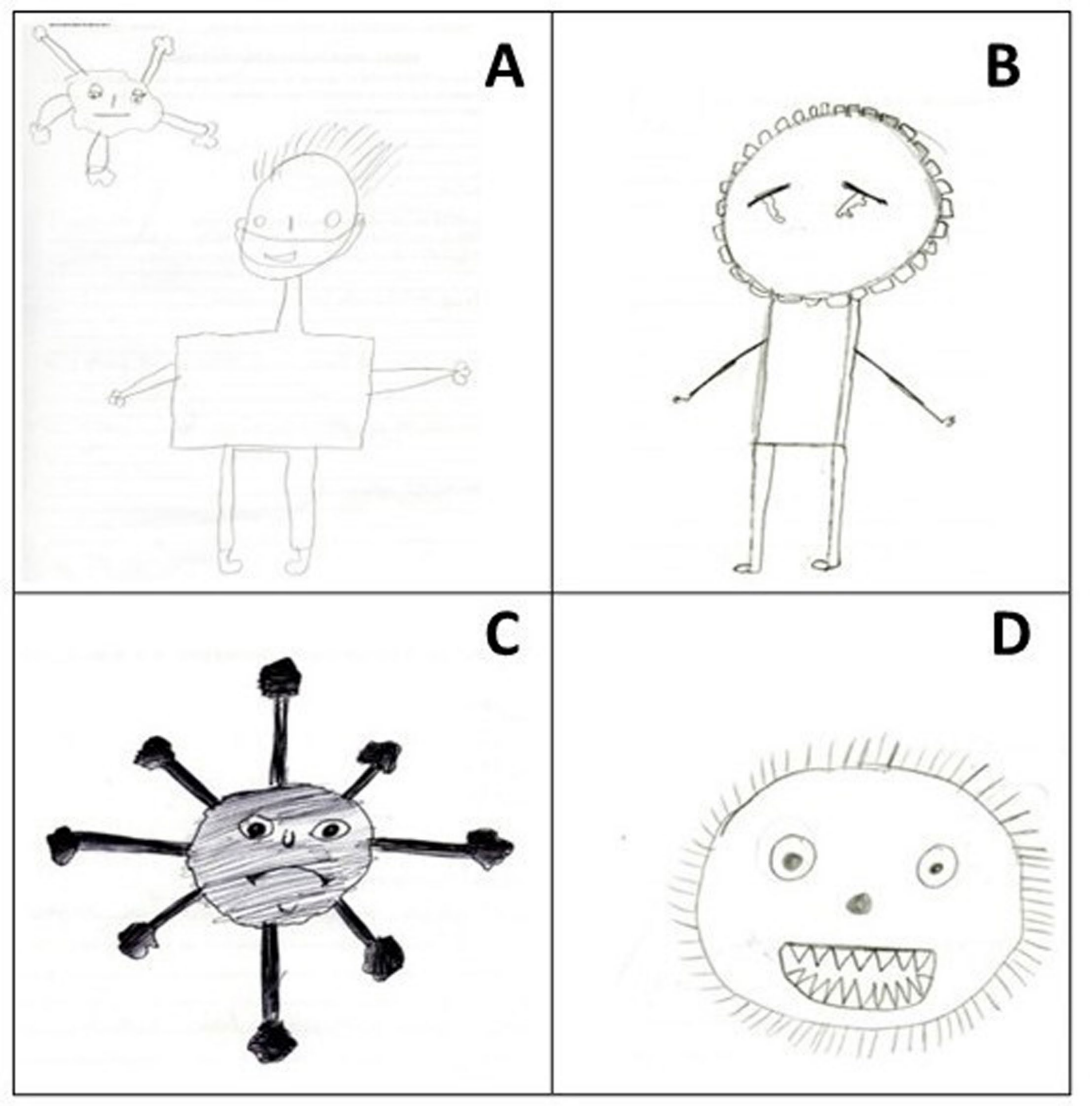
Four children’s drawings made in response to the trigger term “coronavirus,” included in the subcategory “emotion”: “apathy” **(A)**, “sadness” **(B)**, “aggressiveness” **(C,D)**.

The “Emotions” category under the same theme “actions aimed at protection” comprises four subcategories: “apathy,” “sadness,” “aggressiveness,” and “happiness” (Table 2; Figure 5).

The element of apathy was evident in the drawing in Figure 5A, as reflected in the virus’s “facial” expression. The child who drew this picture may have thought of this idea when considering that the virus shows this emotion because the person it intended to infect was wearing a mask, preventing it from succeeding in its goal of infecting him. Furthermore, the sadness emotion is evident in the drawing in Figure 5B, as shown in the infected person’s facial expression.

The two lower drawings (Figures 5C,D) illustrate the virus’s aggressiveness, suggesting it is a harmful organism that spreads by infecting people, causing illness and, in some cases, death. In Figure 5C, these traits are conveyed by the angry/furious look the child attributed to the virus; in Figure 5D, this aggressiveness is evident in the virus’s sharp teeth, ready to attack and infect people, and in its eyes, which appear to show euphoria. These elements in the drawings reflect a mixture of emotions that recall the students’ perceptions of the coronavirus. These data corroborate the study by Idoiaga and collaborators (33), who analysed Spanish children’s SR and emotions about the COVID-19 pandemic. The aforementioned study identified emotions of fear and concern about contracting the virus, guilt if they infected others, fear, nervousness, loneliness, sadness, boredom and irritation, as well as security, calm and happiness. Based on these findings, it is clear that the COVID-19 pandemic context has elicited a range of feelings and emotions in children.

Although the present study shows points of convergence with the work of Idoiaga and collaborators (33), important conceptual and methodological distinctions highlight its relevance and contribution to the field of health education. The Spanish study focused on children’s immediate emotional and symbolic responses during the confinement period, whereas the present work, by adopting the structural approach to SR, enabled the identification of elements that stabilised in children’s cognitive structure over time, since it was conducted several years after the peak of the pandemic. Furthermore, the findings provide a basis for planning health education and life skills interventions by allowing a deeper understanding of how pandemic-related meanings are reorganised, stabilised, and pedagogically mobilised within the sociocultural context investigated.

**Figure 6:**
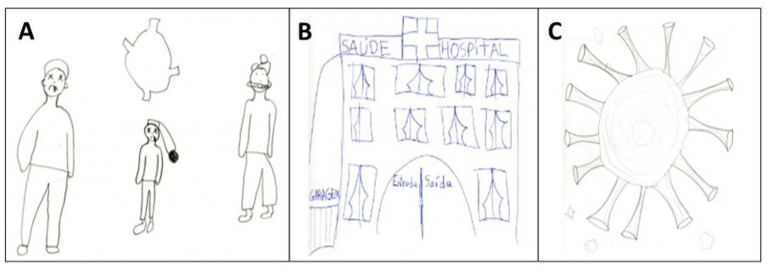
Three children’s drawings made in response to the trigger term “coronavirus,” included in the subcategory ‘contamination’ category: “death,” “hospital,” “virus,” “droplets,” and “illness”.

The second theme, “Lack of care”, includes the category’ contamination,’ which in turn includes five subcategories: “death”, “hospital”, “virus”, “droplets”, and “illness”, with the virus the most representative, with a total of 19 elements evident in the children’s drawings (Table 2; Figure 6).

Figure 6A illustrates the most serious consequence of Coronavirus infection, “death.” According to the child’s explanation, the person on the right is the only one wearing a mask and is alive; the other two were not wearing masks and are dead, evidencing the protective measure. Interestingly, this child’s message demonstrates awareness that viral contamination can be fatal. This notion is common among children, as Sousa and collaborators (34) reported that 56% of 2 to 11-year-old children mentioned death when asked what happens to a person who contracts coronavirus.

As a note, not everyone who becomes infected with coronavirus develops COVID-19 (35). This distinction between what the virus is and what the disease is was not observed in the present study, which analysed specific evocations and drawings made in response to the trigger term “coronavirus.”

Figure 6B shows a hospital. It can be inferred that the child associated this space with a reference centre for treating people who contracted the virus at some point and therefore needed medical care.

Finally, Figure 6C illustrates the coronavirus, which is somewhat faithful to the virus’s structure as widely depicted in the media and reflects what was observed in most of the children’s drawings. Indeed, the children’s drawings reflected an attempt to visually reproduce the coronavirus as commonly represented in the media, particularly its spherical shape and tubular structures, except for size proportions, which are, of course, submicroscopic.

In view of the analyses presented herein, it should be noted that the possible central core of the children’s group SR is formed mainly by the elements “illness,” “death,” and “virus,” which were somewhat representative and were verified in the three analysis procedures. From the perspective of SR, social groups elaborate shared systems of meaning that enable them to interpret unfamiliar phenomena and integrate them into everyday thinking (6). This convergence suggests that these elements occupy a prominent position in both children’s verbal expressions and their visual representations of the coronavirus. According to the structural approach to SR (13), the central core organizes the system of meanings associated with the representational object and provides stability to the representation by defining its fundamental significance. In this sense, the recurrence of these elements in children’s responses suggests that they may structure how the pandemic is interpreted by this group.

Although the element “illness” was not explicitly mentioned in the children’s drawings, it was implicitly present through visual references to contamination, hospitals, and death, indicating that drawings tend to emphasise experiential and contextual aspects of the representation rather than abstract labels. This figurative pattern should be interpreted in light of the strong mediation of adult discourse and public communication during the pandemic, processes through which SR are produced and circulated in society (6).

The identification of the aforementioned elements in the drawings illustrates the process of objectification described by Moscovici (6), through which abstract or unfamiliar concepts are converted into concrete images. In the data presented, this process materializes in the figurative representations of the virus, where the invisible microorganism takes on familiar contours and shapes, integrating itself into the symbolic and everyday universe of the children and contributing to the formation of a figurative nucleus of the representation.

Data triangulation further supports the interpretation of “masks” as a possible element of the central core of children’s SR. In the CA, this element appeared explicitly in five of the children’s drawings, indicating its relevance in their symbolic elaborations of the pandemic. In the similarity analysis, “masks” assumed a centralising role, showing strong connections with other elements and occupying a structurally strategic position within the representational network. From the perspective of the structural approach to SR, elements located in the second quadrant of the Vergès diagram—as observed for “masks” in this study—may reflect either central elements for specific subgroups or elements belonging to the peripheral system that are undergoing a process of centralisation. As proposed by Abric (13), elements located outside the central core, particularly in the peripheral system, may acquire greater organising power over time and potentially integrate the central core as the representation stabilises. Taken together, the evocation task and the drawings function in a complementary manner: while evocations highlight the salience of symbolic elements circulating in social communication, drawings reveal how these elements are concretely experienced and re-signified in children’s everyday lives.

One factor that stood out in the analyses was the absence of any mention of the vaccination process, given that the data collection (2024) occurred after the nationwide implementation of the vaccine. This absence suggests the limited symbolic relevance of vaccination within the structure of children’s SR, as references to this process did not appear in either the central core or the peripheral system. In studies based on prototypical analysis of free evocations, the presence, frequency, and salience of elements provide important indicators of the representational structure, making the absence of certain themes analytically meaningful when interpreting the representational field (16). A plausible reason for this is that, at the peak of the pandemic, between 2020 and 2021, these children were very young, aged 6 to 9, and had not yet been vaccinated. Beyond an age-related explanation, this pattern may indicate that vaccination was not incorporated into children’s experiential universe, as it was largely framed as an adult-controlled, externally determined practice.

This finding contrasts with studies that have identified vaccination as a salient element in SR related to COVID-19 among adults, often associated with themes such as mistrust, fear of adverse effects, and misinformation (36). In this sense, the limited presence of vaccination in children’s representations may also point to opportunities for health education initiatives in school contexts. By promoting discussions about vaccines, their safety, and their role in disease prevention, educational actions may contribute to strengthening children’s understanding of vaccination as a scientifically grounded and socially relevant health practice, while also countering the circulation of misinformation surrounding immunization processes.

An important consideration is the temporal distance between the pandemic peak and the time of data collection. Conducting the study at a later stage of the health crisis enabled analysis of which elements became embedded in children’s cognitive structure, manifesting in their SR. This decision is consistent with the perspective of SR Theory, which understands meanings as being progressively reorganised and consolidated through social communication and lived experience. Thus, the elapsed time constitutes both a limitation, considering that it may attenuate certain contextual details, and an analytical opportunity to identify the elements that persisted and became structurally significant in children’s representations.

We also highlight the significant role of mass media in disseminating information. On the one hand, the media played a crucial role in disseminating strategies for combating the virus and encouraging protective measures, thereby contributing to health promotion. On the other hand, the visual representations shared through the media helped create a visual identity for the virus, as reflected in the drawings of many children. At the same time, this intense exposure to media images may have reinforced fear-centred and biomedical framings of the pandemic, limiting the circulation of meanings related to participation, care, and collective agency.

Finally, it is worth highlighting the predictability of the findings obtained. As discussed previously, the media played a central role in shaping children’s meanings about the coronavirus, which makes the identification of the terms “illness,” “death,” “virus” and “masks” as possible elements of the SR central core of the group analysed understandable. These findings indicate that the central core is structured predominantly around biomedical and threatening elements, rather than preventive elements or those related to action and care. These findings point to the need for school-based interventions that intentionally promote action-oriented, participatory, and reflective approaches to health education, capable of reconfiguring children’s representations beyond fear and biomedical threat.

The structuring of the possible central core of the SR becomes concerning insofar as such elements do not necessarily promote the development of life skills. This reflection is particularly relevant in light of the structural approach to SR, in which the central core organises meanings and guides social practices, potentially influencing how children understand, experience, and respond to health-related issues.

## 4 Final considerations

The COVID-19 pandemic, triggered by the coronavirus (SARS-CoV-2), sparked a wave of insecurity and fear amid the limited information available at the time. The global spread of the virus and its potential for contamination led to significant changes in social dynamics, directly impacting people’s daily lives. Among the initial biosafety measures was social isolation, which confined the population to their homes, with media reports being their primary source of information and guidance.

In this context, health education and life skills development emerged as a crucial tool for enhancing communication between health professionals and citizens, thereby facilitating the development of educational and health promotion initiatives. Moreover, the pandemic period has emerged as a crucial area of research, particularly in understanding its impacts on mental health. The loss of physical contact and the pervasive fear of virus transmission have been recurring themes. Children, especially, have been significantly affected, having to distance themselves from their peers and educators, often within an unstable family environment. The way children perceive and construct their understanding of the coronavirus is pivotal, as it can influence educational health actions and decision-making. It underscores the critical importance of investigating their SR of the coronavirus, which is the primary focus of this study.

Thus, the SR of Portuguese children regarding the term “coronavirus” were investigated to understand what they understand about the topic and identify possible gaps in these representations. The results showed children’s knowledge of ways to protect themselves from coronavirus contamination, as well as the notion that it can lead to illness and death. Children presented a visual representation consistent with the broad virus’s composition expressed through drawings, which can be explained by the role of the mass media in spreading this information.

The data obtained allow us to infer that the probable central core of the social representations of the coronavirus among the Portuguese children who comprised the investigated group is structured around biomedical and threat-related elements, more specifically, the terms “illness,” “virus,” “death,” and “masks.” These elements were consistently observed across the three analyses conducted and reflect the influence of social communication and lived experiences during the pandemic, revealing how children symbolically organised their understanding of the crisis. From an educational perspective, recognising these representational structures is essential for planning health education interventions that move beyond risk-centred narratives and promote life skills, critical thinking, and active participation in health-related practices.

In view of the discussions presented, it can be concluded that the children’s group investigated holds SR about the coronavirus that are influenced by scientific knowledge and may result from health education and life skills campaigns conveyed by the media during the COVID-19 pandemic. These campaigns, which are important mechanisms for constructing and sharing representations, can empower children to make informed decisions about their health and safety.

An important aspect to highlight is the particularity of this study’s findings. In this regard, it is worth noting that research grounded in SR does not aim at generalisation, since representations are constituted through social interactions situated within specific sociocultural contexts. Nevertheless, the findings presented here are relevant insofar as they provide insight into how a specific group of children elaborated, organised, and re-signified meanings about the coronavirus within a defined historical and territorial context, revealing which elements became more persistent and structurally significant in their social representations over time. Although the findings are not generalisable, they offer important analytical contributions to understanding processes of symbolic construction in educational contexts.

These results reinforce the importance of school health education combined with the media disseminating scientific and health information. Results also underscore the importance of identifying children’s SR regarding the coronavirus, as this can significantly inform the development of health education and the construction of scientific knowledge about viruses and diseases. Finally, this study can inspire and guide future work focused on children’s health education and life skills development.

## Data Availability

The original contributions presented in the study are included in the article/supplementary material, further inquiries can be directed to the corresponding author.
